# The peacebuilding potential of healthcare training programs

**DOI:** 10.1186/s13031-016-0096-3

**Published:** 2016-09-13

**Authors:** Kyle G. Ratner, Lindsay B. Katona

**Affiliations:** 1 Department of Psychological and Brain Sciences, University of California, Santa Barbara, Santa Barbara, CA USA; 2 Santa Barbara Cottage Hospital, Santa Barbara, CA USA

**Keywords:** Healthcare training, Peacebuilding, First aid, Health as a bridge for peace

## Abstract

Global health professionals regularly conduct healthcare trainings, such as first aid courses, in disadvantaged communities across the world. Many of these communities lack healthcare infrastructure because of war and political conflict. The authors draw on their experience conducting a first aid course in South Sudan to provide a perspective on how healthcare trainings for people with no medical background can be used to bridge ethnic, political, and religious differences. They argue that a necessary step for turning a healthcare training into a vehicle for peacebuilding is to bring people from different communities to the same physical space to learn the course material together. Importantly, simply encouraging contact between communities is unlikely to improve intergroup relations and could be detrimental if the following features are not incorporated. Buy-in from respected community leaders is essential to ensure that training participants trust that their safety during the training sessions is not at risk. Trainers should also create a supportive environment by conferring equal status and respect on all trainees. Finally, hands-on training exercises allow for positive interactions between trainees from different groups, which in turn can challenge stereotypes and facilitate cross-group friendships. These features map onto social psychological principles that have been shown to improve intergroup relations and are consistent with lessons learned from peace through health initiatives in public health and medicine. By adopting peacebuilding features, healthcare trainings can serve their primary goal of medical education and provide the added benefit of strengthening social relations.

In many locations around the world, war and intergroup conflict have destroyed healthcare infrastructure and have rendered large segments of the population vulnerable to disease and injury. A shortage of medical services and healthcare professionals in these regions often means that community members without medical training are the default first responders. The World Health Organization (WHO) has advocated for an expansion of prehospital training courses, such as short courses in first aid, to empower lay people all over the world with easy to learn lifesaving knowledge and skills to help themselves and others when emergencies arise and healthcare professionals are not readily available [1]. Although the medical value of these training programs is clear, such trainings also have valuable and untapped peacebuilding potential.

Our perspective on this issue comes from conducting a small research project in South Sudan. We were part of a group, the Maine-African Partnership for Social Justice (MAPSJ), that investigated whether training in wilderness first aid techniques could increase medical knowledge in South Sudanese villagers with minimal access to healthcare resources. Although we provide a comprehensive account of our research elsewhere [2], in brief, 50 villagers participated in a multi-day training course held in the Kit Region of South Sudan. The course was a mixture of lectures and hands-on sessions that taught basic first aid principles and how to use everyday objects in one’s environment to dress wounds, treat dislocated joints and fractured bones, and transport people with injuries. Villagers from across the region were brought together to take the course. Participants were from different tribes, spoke different languages, and practiced different religions, but they all had the same desire to learn basic medical information and techniques.

Our work focused on assessing the effectiveness of the wilderness first aid training, and it did not occur to us during planning that our project might have peacebuilding benefits. However, at the conclusion of the training, several participants told us that the opportunity to interact with people from other villages in a supportive setting allowed them to forge new relationships that they thought would strengthen community bonds. The collaborative energy generated by the training was also evident in spontaneous discussions among participants about how the villages could pool resources and work together to lobby the local government to improve healthcare for their region. Robert Owot, one of the South Sudanese men who was instrumental in organizing the first aid training, a parallel labor and delivery training, and another effort to bring a regional school to the area, wrote a letter to our group in which he said, “This region inhabits four tribes that have been living indifferently for decades, but the health trainings initiated by MAPSJ, which have been introduced to these people without discrimination, have improved relations and co-existence greatly amongst the people.”

In hindsight, we wish we had collected empirical data to support the anecdotal indications that our training facilitated positive interactions that helped people transcend group boundaries. Despite the absence of data, this experience inspired us to reflect on the features of our program that might have had a positive social impact on these villagers. In what follows, we highlight several observations and attempt to connect them to foundational social psychological theory on improving relations between groups and also lessons learned from the WHO Health as a Bridge for Peace (HBP) program and similar peace through health initiatives. We believe that our observations add to the existing peace through health literature because the majority of this work has focused on healthcare professionals working in conflict areas and not healthcare trainings for lay people who primarily have other professional roles. To be clear, our goal is not to provide a comprehensive analysis or make strong claims about the peacebuilding effectiveness of our particular program. Such assertions would require further research and long-term assessment. Instead, we hope that sharing our experience motivates other organizers of healthcare trainings for lay people in regions of conflict to consider how their programs can improve medical knowledge and simultaneously strengthen community relations.

There were several features of our training program that we believe facilitated positive interactions among members of neighboring villages. The first feature was the decision to bring members of multiple villages together to the same location for a single training, instead of the more typical approach of conducting separate trainings for people of different groups. Our rationale for conducting a single training was practical. It was more cost effective, allowed the training to reach more participants, there was relative stability in the area, and our South Sudanese partners encouraged the approach. However, bringing people together to the same location proved more valuable than we anticipated because it served as a necessary condition for members of various villages to interact and have positive social contact.

It was also critical that our training filled a medical need. Because the training offered skills and knowledge that were novel to participants and valued by the local community, the village leaders sanctioned our training program and encouraged people to participate. This buy-in from respected members of the local community was crucial because it provided trainees with reassurance that the training was worthwhile and also reduced the perceived risk of interacting with previously unknown participants from neighboring villages.

Several features of our program helped create a psychologically safe environment and allowed participants to feel personally invested in the experience. First, we did our best to treat members of all villages equally, which signaled respect and equal status. Additionally, we provided participants with a voice to shape their educational experience. At the end of each day, we asked the trainees what they liked and did not like about the training sessions and adjusted the lessons for the next day accordingly. We did this to maximize the relevance of the training to the participants. However, we think that this hierarchy flattening dialogue also increased the trainees’ investment in the program because it communicated that we were interested in what they wanted to get out of the training and were not just talking at them.

Participants also seemed to give special value to the name badges and other materials we gave them as part of the training. The purpose of the name badges was to help everyone learn each other’s names, but it is possible that these badges provided a visual cue of their new shared identity “first aid trainees,” which helped trainees see themselves and others as a group with a common purpose.

Finally, hands-on training exercises put people in close physical contact with members of other villages. In these exercises, some trainees role-played various injuries while others worked together to provide the recommended treatment. Working together provided moments of laughter, physical touch, exchange of ideas, and helping each other – humanizing experiences that create social connections and challenge stereotypes.

These features of our training program emerged organically, but it is notable that they resemble social psychological principles that have long been thought to promote positive relations between groups. For instance, classic theorizing by the psychologist Gordon Allport highlighted how intergroup interactions benefit from the support of authority figures, equal status between groups, common goals and superordinate identities, and intergroup cooperation [3]. The importance of these factors for facilitating positive intergroup contact has been empirically supported by a recent meta-analysis [4]. Other influential social psychological work has demonstrated the value of giving people a voice in their experiences. This work indicates that when everyone is treated equally and given a voice to express their opinions and concerns, people are motivated to identify with the group that is affording interpersonal respect and also engage in cooperative behavior [5].

The public health and medical literature on peacebuilding through health, which we will collectively refer to by the WHO moniker “Health as a Bridge for Peace (HBP),” provides further clues about how basic first aid trainings might be used to promote peace. The critical insight of the HBP approach is that healthcare and peace are often intrinsically linked. This is the case because discord between groups can cause disparities in healthcare, injuries from violence, mental health problems, and conditions for infectious disease to spread. At the same time, health professionals can promote peace in a number of ways, including reframing conflicts in public health terms, leveraging medical care and resources to promote ceasefires, and providing mental health treatment to heal the psychological trauma of war [6]. As with the social psychological literature, the importance of building relationships through respect, equal status, and giving a voice are emphasized by HBP theorists as critical for bringing people together [7]. Medical trainings figure prominently in the HBP literature, but the focus is primarily on trainings of healthcare professionals. Evidence from the Balkans to the Middle East has shown that bringing together healthcare professionals from differing sides of a conflict for trainings and discussions can improve both regional healthcare services and cross-group cooperation [8, 9]. To our knowledge, very little HBP work has examined how healthcare trainings for people without medical backgrounds can serve as a bridge for peace. This likely reflects the reality that many of the practitioners involved in basic first aid trainings around the globe are not part of the HBP community. Yet, principles from HBP trainings for medical professionals are very translatable to first aid trainings for non-healthcare professionals and provide a useful guide for best practices.

It is also possible that healthcare trainings for lay people may have peacebuilding advantages over conventional conflict resolution interventions, particularly when groups participating in the trainings are not actively seeking intergroup dialogue. The reason for this is that unlike conflict resolution programs that emphasize direct discussion about conflicts, participants in healthcare trainings are not required to discuss these difficult issues. Talking about disagreements and injustices can be aversive, especially when people anticipate and experience confrontation. This uneasiness might cause people to avoid conflict resolution programs that involve such discourse. In contrast, people in healthcare deprived regions are highly motivated to participate in healthcare trainings for the medical knowledge. Because these trainings focus attention on the medical skills and information that can be acquired, the social interactions are in the background. These social interactions, however, provide an important opportunity for conversation to occur among people of different groups. A common interest in healthcare creates a built-in topic for trainees to discuss that can make the conversation pleasant and fluent. As the conversation turns to small talk, people might uncover similarities that they did not know about each other. The rapport established in these non-confrontational interactions can increase feelings of social connection and mutual respect, which can be invaluable if future discussions involve direct dialogue about intergroup tensions.

Healthcare trainings that bring groups of people together might not always be feasible and could backfire in certain circumstances, such as when groups are engaged in intense conflict. It is notable that the region of South Sudan where we conducted our training was not embroiled in violence. In areas of active fighting, buy-in from community leaders might be difficult to obtain and potential trainees might be fearful of participating. As a result, healthcare trainings might be most effective at promoting peace when used preventively to increase social connections between groups that are at risk for conflict but currently coexist without trouble, or reparatively in post-conflict regions and refugee resettlement areas, particularly when the raw emotions of war have begun to dissipate.

It is also important to recognize that well meaning interventions can sometimes have a net negative effect on the communities they are designed to help [10]. It is currently unknown whether incorporating peacebuilding features into healthcare trainings could have detrimental and unintended consequences. As a result, people conducting such efforts should pay particular attention to any concerns expressed by trainees and other community members.

Our hunch is that many global health professionals who are designing healthcare training programs for lay people in low and middle-income communities are focused on the public health objectives and have not contemplated how their programs can also build cross-group trust and friendships. However, we urge these individuals to carefully consider whether their programs can be used to promote peace. We also hope that HBP practitioners and researchers will further develop the ideas we have proposed and rigorously assess their utility. Given that healthcare trainings are regularly conducted all over the world, structuring them to promote peace could have large and multiplicative benefits on the stability of communities around the globe.
